# Overexpression of SPARC gene in human gastric carcinoma and its clinic–pathologic significance

**DOI:** 10.1038/sj.bjc.6602213

**Published:** 2004-11-23

**Authors:** C-S Wang, K-H Lin, S-L Chen, Y-F Chan, S Hsueh

**Affiliations:** 1 Department of General Surgery, Chang Gung Memorial Hospital at Chiayi, Taiwan; 2 Department of Biochemistry, Chang Gung University, 259 Wen-hwa 1 Road, Taoyuan, Taiwan; 3 Department of Pathology, Chang Gung University, 259 Wen-hwa 1 Road, Taoyuan, Taiwan

**Keywords:** gastric cancer, SPARC, real-time RT-PCR, microarray

## Abstract

Gastric cancer is the second most common cancer in the world and the fifth leading cause of cancer-related death in Taiwan. To improve the survival of gastric cancer patients, biomarkers for early detection and effective anticancer therapy are required. An essential first step is to profile gene expression in gastric cancer and identify genes that are aberrantly expressed, and to do this cDNA microarrays were performed. The clinic–pathologic correlation and prognostic significance of the aberrantly expressed genes were evaluated to identify novel biomarkers of gastric cancer. Fresh surgical samples of tumour tissue and matching noncancerous mucosa were obtained immediately after gastric resection in 43 patients. Secreted Protein, Acidic and Rich in Cysteine (SPARC) (Osteonectins), one of the most highly expressed genes in both intestinal and diffuse gastric cancers in our microarray results, was selected for further study. The overexpression of SPARC was verified using real-time quantitative-reverse transcription–polymerase chain reaction (Q-RT–PCR), Northern blot and immunohistochemical staining. The expression of SPARC in tumour tissues was, on average, 4.27-fold increased (95% CI 2.68–5.85) compared to adjacent noncancerous mucosa (*P*<0.001). The expression of SPARC was higher in advanced (T2, T3 and T4) cancer compared to the early (T1) cancer (*P*=0.048) with regard to depth of wall invasion. Higher expression of SPARC was significantly associated with lymph node metastasis (*P*<0.001), lymphatic invasion (*P*=0.004) and perineural invasion (*P*=0.047). Expression of SPARC in patients in stage II and above was significantly higher than those in stage I (*P*=0.017). The 3-year survival of patients with lower expression of SPARC was significantly better than those with a higher expression (log rank *P*=0.047). These data indicate the potential of SPARC as a prognostic marker for gastric cancer.

Gastric cancer is the second most common cancer in the world, while in Taiwan it is the fifth leading cause of cancer-related death. Surgery remains the only cure for this disease. In a recent study, more than 30% of surgical patients were too advanced to receive curative resection (Wang *et al*, 2002). To improve the poor survival outcome and permit earlier diagnosis, there is a need for new prognostic indicators or tumour markers more sensitive than those currently available, including CEA, CA19-9 and others (Marrelli *et al*, 1999).

Histomorphologically, gastric cancer is divided into two main types, ‘intestinal-differentiated’ and ‘diffuse-undifferentiated’ (Lauren, 1991; Tahara, 1993). However, lesions with a similar type may differ in biological aggressiveness and response to therapy. The molecular events for development and progression of gastric cancer are a complex process involving multiple genes and multiple steps, sequentially or in concert (Tahara, 1993). Several risk factors including genetic alterations, chromosomal instability, and *Helicobacter pylori* infection have been reported for gastric cancer (Barreto-Zuniga *et al*, 1997; Yasui *et al*, 2001; Kim *et al*, 2003). Moreover, numerous biomarkers identified have contributed to our knowledge of the molecular or cellular mechanisms of gastric carcinogenesis and progression (Yasui *et al*, 2001). Most biomarkers are prognostic factors used to indicate the groups of patients at risk of relapse or metastasis (Allgayer *et al*, 1997). However, useful biomarkers for early detection and therapy are still lacking and depend on future studies. Gene expression profiling offers a new approach for cancer diagnosis and may contribute to our understanding and the future elucidation of a cure (Pusztai *et al*, 2003; Ransohoff, 2003). To achieve these goals, the relevant subsets of differentially expressed genes of interest must be identified, cloned and studied in detail. Complementary DNA (cDNA) microarrays are currently recognised as a powerful technique to search for novel biomarkers through the establishment of gene expression profiles (Ransohoff, 2003).

Using cDNA microarry, we identified a highly expressed gene for gastric cancer, Secreted Protein, Acidic and Rich in Cysteine (SPARC), also designated as osteonectin and BM40 (Motamed, 1999). SPARC is a single-copy gene mapped to mouse chromosome 11 and to the long arm of human chromosome 9 (Motamed, 1999). High conservation is observed between the bovine, mouse and human SPARC sequences. The SPARC gene expresses a major mRNA species 2.2 kb in length, which is translated into a 43-kDa Ca^2+^-binding glycoprotein. Initially in 1981, SPARC was described as a bone matrix protein involved in bone mineralisation. Recent studies have revealed other biological functions including cell proliferation (Sage *et al*, 1995), migration, morphogenesis (Strandjord *et al*, 1995), deadhesion, antiproliferation (Motamed, 1999; Bornstein and Sage, 2002), differentiation (Bassuk *et al*, 1999) and angiogenesis (Kupprion *et al*, 1998). SPARC was reported to be overexpressed in a variety of human malignancies, including melanoma, glioma, meningioma, colorectal, breast, oesophageal, renal cell, prostate, bladder and hepatocellular carcinoma (Porte *et al*, 1995; Le Bail *et al*, 1999; Massi *et al*, 1999; Menon *et al*, 2000; Takano *et al*, 2000; Thomas *et al*, 2000; Sakai *et al*, 2001; Yamanaka *et al*, 2001; Iacobuzio-Donahue *et al*, 2002; Yamashita *et al*, 2003). However, SPARC was repressed in ovarian cancer (Paley *et al*, 2000).

Overexpression of the SPARC gene was observed in human gastric cancer in two other reports (Wewer, 1988, Maeng *et al*, 2002b). However, both studies had no detail in clinic–pathologic correlation. Overexpression of SPARC gene was also observed in gastric adenocarcinomas and adenomas induced by a chemical carcinogen (*N*-methyl-*N*′-nitro-*N*-nitrosoguanidine) in Lewis and WKY rats (Maeng *et al*, 2002a). It implied that SPARC expression appeared in the initiation stage of experimental gastric carcinogenesis (Maeng *et al*, 2002a).

The aim of this study was to identify useful biomarkers using cDNA microarrays, and to investigate their clinical significance in gastric cancers. We found that SPARC expression was significantly higher in the advanced stage of gastric cancer in comparison to the early stage, indicating SPARC potential as a marker for the more advanced forms of gastric cancer.

## MATERIALS AND METHODS

Between September 2000 and November 2001, a total of 43 patients with gastric cancer entered this study. They included 24 male and 19 female patients. The median age was 61 years (range 35–83). All patients had undergone gastric resection including 16 total gastrectomies and 27 subtotal gastrectomies.

### Clinic–pathologic studies

Re-sected specimens were studied pathologically according to the criteria described in the Japanese General Rules for Gastric Cancer Study (Japanese Gastric Cancer, 1998) and the UICC pTNM classification (Sobin and Fleming, 1997). The study items included age, gender, tumour location, tumour size, gross (Borrmann) type, wall invasion, resection margin, histologic type, lymph node metastasis, vascular invasion, lymphatic invasion and perineural invasion. The histological features were classified into two types: (1) intestinal or differentiated type, consisting of papillary and/or tubular adenocarcinomas and (2) diffuse or undifferentiated type, consisting of poorly differentiated, signet-ring cell, and/or mucinous adenocarcinomas (Japanese Gastric Cancer, 1998; Tahara, 1993). After discharge, all patients received periodic follow-up in the outpatient department until the time of writing, or until their death.

### Tumour samples

Fresh samples of both tumour tissue and adjacent noncancerous mucosa were obtained immediately after gastric resection. The samples were carefully dissected from resected specimens by a pathologist, and immediately snap frozen in separate vials using liquid nitrogen. These frozen specimens were stored at −70°C in a tumour bank until use.

### cDNA microarray analysis

Specimens were taken from fresh surgical samples of tumour tissue and matching normal mucosa, stored in a −70°C tumour bank. For fluorescence labelling of cDNA, 30 *μ*g of total RNA from tumour cells and 50 *μ*g of total RNA from normal mucosa cells were reverse transcribed in the presence of Cy3-dUTP and Cy5-dUTP (Amersham Inc., Piscataway, NJ, USA), respectively. Labelled cDNA was purified and resuspended in the hybridisation buffer as described (Eisen and Brown, 1999). For the duplication experiment, we switched the Cy3-dUTP and Cy5-dUTP labelling to the normal and tumour cells RNA, respectively. Human Universo-Chip 8 K cDNA arrays (Asia Bio-Innovations Corporation, Taipei, Taiwan), containing 7597 genes (cDNAs), were used to distinguish the specific gene(s) that were overexpressed or underexpressed in five pairs of human gastric specimens. Hybridised slides were scanned using the GenePix 4000B scanner (Axon Instrument, CA, USA) and images were processed using the GenePix Pro 3.0 (Axon Instrument). Microarray data were analysed using the eGenomix V1.0 (Asia BioInnovations Corporation, Taipei, Taiwan) and EXCEL (Microsoft, Seattle, WA, USA) software. Both samples and genes were classified by a two-way clustering analysis to identify genes that were differentially expressed between cancer and nontumorous tissues.

Among the differentially expressed genes, those that were highly upregulated in both histological types of tumour were selected for further confirmational study including Northern blot analysis, real-time Q-RT–PCR and immunohistochemistry.

### Northern blot analysis

Equal amounts of total RNA (20 *μ*g) were analysed on a 1.2% agarose-formaldehyde gel. The RNA was then blotted onto a nitrocellulose membrane and subjected to Northern blot analysis (Lin *et al*, 2002). A *α*^32^P-dCTP-labeled random-primed probe (3000 Ci/mmol; Amersham) was hybridised to the membrane. The probe was a full-length SPARC cDNA fragment, which was amplified by the PCR. Membranes were subsequently reprobed with a *α*^32^P-labelled glyceraldehyde-3-phosphate dehydrogenase (GAPDH) cDNA fragment to verify an equal application of RNA to each lane.

### Real-time quantitative-reverse transcription–polymerase chain reaction

Total RNA was extracted from cells using Trizol. Subsequently, the first strand of cDNA was synthesised using the Superscript III kit for RT–PCR (Life Technologies, Rockville, MD, USA). Briefly, total RNA was denatured at 65°C for 5 min in the presence of 0.5 *μ*g oligo dT and 1 mM dNTP. After chilling on ice for at least 1 min, RT was allowed to proceed at 25°C for 5 min in the presence of 1 × first-strand buffer, 5 mM DTT and 40 units of RNase inhibitor. The reaction was then allowed to proceed at 50°C for another 60 min. The reaction was terminated by heat inactivation at 70°C for 10 min. Real-time Q-RT–PCR was performed in a 25 *μ*l reaction mixture containing 50 nM forward and reverse primers, 1X SYBR green reaction mix (Applied Biosystems, Werrington, UK) and various amounts of template. The reaction was performed with preliminary denaturation for 10 min at 95°C to activate *Taq* DNA polymerase, followed by 40 cycles of denaturation at 95°C for 15 s, and annealing/extension at 60°C for 1 min. Fluorescence emitted by SYBR green was detected by the ABI PRISM 7000 sequence detection system (Applied Biosystems). The primers are forward, 5-CCTGGAGACAAGGTGCTAACAT-3 and reverse, 5-CGAGTTCT CAGCCTGTGAGA-3. Different amounts of template (16, 8, 4, 2, 1 ng) were used in the same reaction to ensure linear amplification. All PCR reactions were performed in duplicate on the same 96-well plate. 18S RNA was used as an internal control amplified in the same PCR reaction and expressed as 2^−ΔCt^ × *k*, where *k* is a constant and −Δ*C*_t_ is *C*_t(sparc gene)_−*C*_t(18S)_. *C*_t_ is defined as the cycle at which fluorescence is determined to be statistically significant above background. The expression of a specific gene in the tumour tissue was determined using the fold of activation over the matching noncancerous mucosa.

### Immunohistochemistry

Formalin-fixed and paraffin-embedded tissues were examined by immunohistochemistry using the avidin–biotin complex (ABC) method (Hsu *et al*, 1981). A tissue block that included both benign gastric mucosa and an area of carcinoma was chosen from each patient. They were sectioned into 4-*μ*m-thick slides and the paraffin removed. After antigen retrieval, tissue sections were stained with 1 : 50 dilution of anti-SPARC monoclonal antibody (ZYMED lab, CA, USA). The staining process was automated and performed on a Ventana immunostainer. A normal skin sample was included in each assay as a positive control (Hunzelmann *et al*, 1998), and staining without primary antibody was used as a negative control. Cytoplasmic patches of brown colour were scored as SPARC positive. The interpretation of the benign gastric mucosa was based on staining results of the superficial epithelium only, because the parietal cells and chief cells of gastric pits are normally stained strongly with SPARC. Comparisons were made between the intensity of the staining of the carcinoma cells and benign superficial epithelium on the same slide. The negative group consisted of cancer cells with no detectable (−) or only trace staining of SPARC immunoreactivity (+1). The positive group consisted of cancer cells with moderate (+2) or high levels (+3) of SPARC immunoreactivity.

### Statistical analysis

When appropriate, the Mann–Whitney *U*-test or Fisher's exact test was used for between-group comparisons. The relationship between the results of two different examinations was analysed by Spearman's correlation test. Follow-up of the patients was carried out until the time of writing or until the patient died. The cancer-specific survival outcome was expressed by applying the Kaplan–Meier method for all patients excluding those who died from surgical complications. The log-rank test was used to compare the prognostic significance of individual variables on survival. Cox's proportional hazards model was used in a multivariate analysis to identify the independent predictors of survival. A *P*-value of <0.05 was considered statistically significant.

## RESULTS

### Clinical characteristics of the patients

Characteristics of all patients included in this study are listed in Table 1

**Table 1:** Correlation between patient's demographic characteristics and the expression level of SPARC measured by Q-RT–PCR

**Parameters**	**No.**	**SPARC (mean±s.e.)**	***P*-value**
*Age (years)*			
<64	22	3.72±1.10	0.653
⩾65	21	4.86±1.21	
			
*Gender*			
Male	24	3.77±0.94	0.874
Female	19	4.93±1.34	
			
*Location*			
Proximal	9	5.32±1.82	0.228
Middle	13	2.30±0.66	
Distal	20	5.31±1.36	
			
*Size (cm)*			
⩽2.5	12	2.20±0.71	0.165
>2.5	31	5.08±1.02	
			
*Gross type*			
Borrmann 0–2	12	2.32±0.81	0.108
Borrmann 3–4	31	5.04±1.02	
			
*Histology*			
Intestinal	16	3.89±0.90	0.624
Diffuse	27	4.51±1.14	
			
*Depth of wall invasion*			
Early (T1)	10	1.81±0.69	0.048
Advanced (T2–4)	33	5.56±0.97	
			
*Lymph node metastasis*			
Negative	11	0.80±0.16	<0.001
Positive	32	5.48±0.97	
			
*Distant metastasis*			
M0	30	3.89±0.80	0.488
M1	13	5.19±1.87	
			
*Pathologic stage*			
Stage 1	11	1.67±0.63	0.017
Stage 2–4	32	5.18±0.99	
			
*Vascular invasion*			
Negative	37	4.07±0.77	0.824
Positive	6	5.59±3.26	
			
*Lymphatic invasion*			
Negative	15	1.65±0.50	0.004
Positive	28	5.69±1.09	
			
*Perineural invasion*			
Negative	29	3.48±0.86	0.047
Positive	14	5.93±1.58	
			
*Peritoneal seeding*			
Negative	36	4.73±0.91	0.508
Positive	7	1.99±0.60	

. The mean tumour size (maximal diameter) was 4.8±3.2 cm (median 3.5 cm, range 0.9–18 cm). The tumours were located in the proximal third of the stomach in nine cases, the middle third in 13, the distal third in 20 and the whole stomach in one. The histological types consisted of intestinal type in 16 and diffuse type in 27 patients. As defined by the depth of wall invasion, early gastric cancer (T1) was noted in 10 (23.3%) cases (mucosa in four and submucosa in six), while advanced cancer included T2 (muscle proper and subserosa) in four, T3 (serosa) in 25 and T4 (invasion to adjacent organs) in four. Lymph node metastasis was found in 32 cases (74.4%). During operation, peritoneal seeding was found in seven (16.3%) and no liver metastasis was noted in any of our patients. The pathologic stage was distributed as IA in six, IB in five, II in four, IIIA in seven, IIIB in nine and IV in 12.

### Results of cDNA microarray

Microarray results were obtained from five pairs of specimens (tumour tissue and adjacent non-tumorous mucosa) of both intestinal and diffuse histological types in duplicate. There were more than 2.8% (216/7597) distinct genes either upregulated (ratio>2) or downregulated (ratio<0.5) in these array experiments. The numbers of highly upregulated genes were 86 in the intestinal type and 138 in the diffuse type (data not shown). Tables 2

**Table 2:** The five most highly overexpressed genes in the intestinal type of gastric cancer tissues by cDNA microarray

**Name**	**Accession no.**	**UG_LINK**	**Symbol**	**Function**	**Fold activation**
Immediate early response 3	AA480815	Hs.76095	IER3	Apoptosis	6.58
Secreted protein, acidic, cysteine-rich (osteonectin)	H95960	Hs.111779	SPARC	Binding protein/ calcium/collagen	6.53
Protective protein for beta-galactosidase (galactosialidosis)	AA916327	Hs.118126	PPGB	m-esterase/ m-deamidase	6.28
Human secretory protein (P1.B) mRNA, complete cds	N74131	Hs.82961		Cell matrix protein/cell adhesion	5.87
Myosin regulatory light chain, complete cds	AA487370	Hs.180224		Apoptosis	5.56

and 3

**Table 3:** The five most highly overexpressed genes in the diffuse type of gastric cancer tissues by cDNA microarray

**Name**	**Accession no.**	**UG_LINK**	**Symbol**	**Function**	**Fold activation**
Secreted protein, acidic, cysteine-rich (osteonectin)	H95960	Hs.111779	SPARC	Binding protein/ calcium/ collagen	9.68
Interferon-inducible	AA862371	Hs.182241	1-8D	Surface molecule	9.16
Collagen, type III, alpha 1	T98612	Hs.119571	COL3A1	Structure protein	7.62
Carcinoembryonic antigen-related cell adhesion molecule 5	AA130584	Hs.118778	CEACAM5	Antigen	7.42
Ribosomal protein L39	N76229	Hs.177461	RPL39	Structure protein/ protein synthesis	6.16

outline the top five most overexpressed genes for the intestinal and diffuse types, respectively. Among them, SPARC was the most highly expressed gene in the diffuse type and the second most highly expressed in the intestinal type of gastric cancer. Therefore, SPARC was selected for further study.

### Verification of the abundance of SPARC mRNA in gastric cancer tissues

Real-time Q-RT–PCR data for the expression of SPARC were measured by the ratio of SPARC signal in tumour tissue to that in noncancerous adjacent tissues. The mean expression of SPARC was 4.27-fold increased in tumour tissues, as compared to normal (95% CI 2.68–5.85), which indicated a significant overexpression (*P*<0.001, by one-sample *T*-test). The patients were divided into two groups according to the ratio of SPARC expression in the tumour samples, as compared to the adjacent normal tissue. There were 12 (28%) patients in the lower expression groups (⩽1.0-fold), and 31 (72%) patients in the higher expression groups (>1.0-fold). The real-time Q-RT–PCR assay yielded very reproducible results that supported the microarray data.

**Figure 1:**
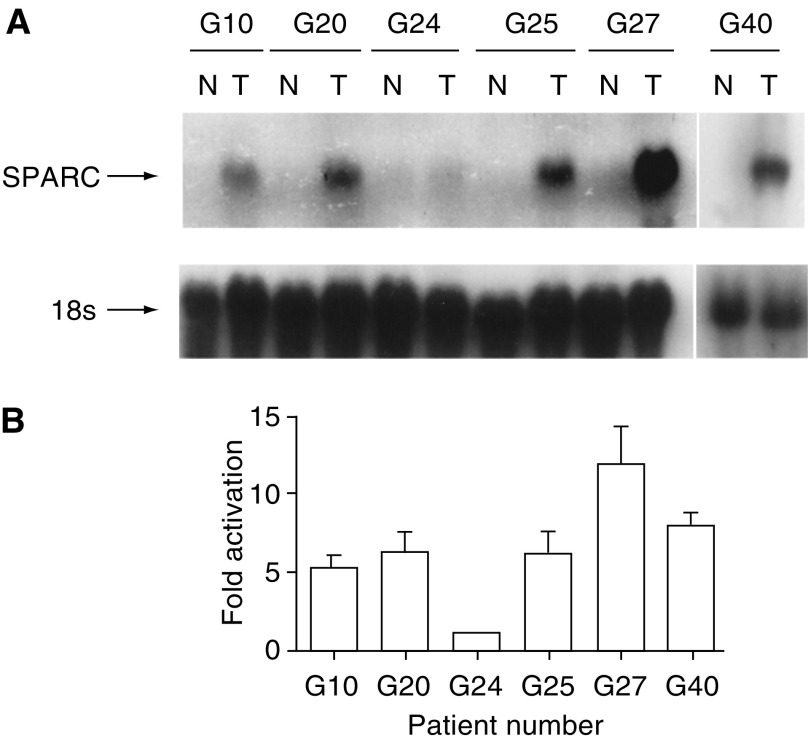
Overexpression of *SPARC* in gastric carcinoma. (**A**) Northern blot analysis revealed a 2.2-kb *SPARC* transcript in cancer tissues examined from our surgical specimens. *SPARC* is overexpressed in all tumour tissues (T) compared to the matched noncancerous adjacent mucosa (N). G10, G20 and G24 are intestinal tumour samples, while G25, G27 and G40 are diffuse-type of tumours. (**B**) The intensities of SPARC expression on the blots shown in (**A**) were quantified, and the extent of activation of SPARC expression was determined at each time point. Fold activation indicates the ratio of SPARC signal in tumour to that in normal adjacent tissue. Data are means±s.e. of values from three independent experiments.

We further confirmed the *SPARC* expression levels in several patients by Northern blot analysis. Figure 1

illustrates *SPARC* expression in several representative patients. A 2.2-kb *SPARC* transcript was detected in all cancer tissues examined. The cancer tissues from both intestinal (G10, G20, G24) and diffuse (G25, G27, G40) types all highly expressed *SPARC* compared to the matched noncancerous adjacent mucosa (Figure 1A and B). The Northern blot analysis determined the mean expression of SPARC in cancer tissues was 4.7-fold (range 1–12) greater than in their noncancerous counterpart, which confirmed the trend observed in the real-time Q-RT–PCR.

### Overexpression of SPARC protein in gastric cancerous tissues demonstrated by immunostaining

**Figure 2:**
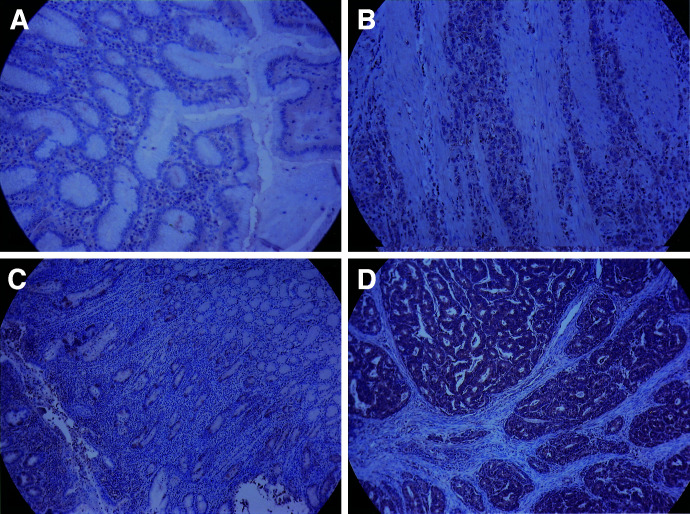
Immunohistochemical staining of SPARC expression in two (G25 and G20) representative human gastric cancer tissues and matching noncancerous mucosa. G25 and G20 are diffuse-type gastric cancer and intestinal-type gastric cancer, respectively. (**A**) G25 noncancerous mucosa, (**B**) G25 diffuse-type gastric cancer, (**C**) G20 noncancerous mucosa, (**D**) G20 intestinal-type gastric cancer. Positive staining of SPARC is indicated by a dark brown colour. The SPARC expression was stained mainly in the gastric cancer cells, and less intensively in the stroma cells.

To investigate the expression and location of SPARC in tissues, immunostaining was performed on gastric cancer tissues and matched noncancerous muscosa. Figure 2B

shows a diffuse-type and Figure 2D an intestinal-type tumour. In contrast, Figure 2A and C are their normal counterparts. The immunostaining was most marked (dark brown colour) in the cancer cells, and low levels were observed in the stroma cells or fibroblasts in gastric cancer tissues. Weak staining was observed for SPARC in the normal gastric epithelial cells (Figure 2A and C). Only a trace of immunoreactivity was distinguished in the adjacent noncancerous mucosa (Figure 2A and C). Among the 40 patients studied by immunohistochemistry, the intensity of immunostaining in tumour tissue was determined as 1+ in 15 patients (37.5%), 2+ in 19 (47.5%) and 3+ in 6 (15%). The higher expression group (2+ and 3+) accounted for 62.5% (25/40). In contrast, the intensity of immunostaining in nontumorous mucosa was 1+ in 35, and 2+ in five. Furthermore, the immmunoreactivity in the cancerous tissues was greater than in the nontumorous counterpart in 23 patients, and equal in 17. The SPARC expression levels in tumour tissue, as determined by immunohistochemistry, was comparable to that ascertained by real-time Q-RT–PCR (Pearson's correlation coefficient=0.421; *P*=0.008).

### SPARC expression and clinic–pathologic correlation

**Figure 3:**
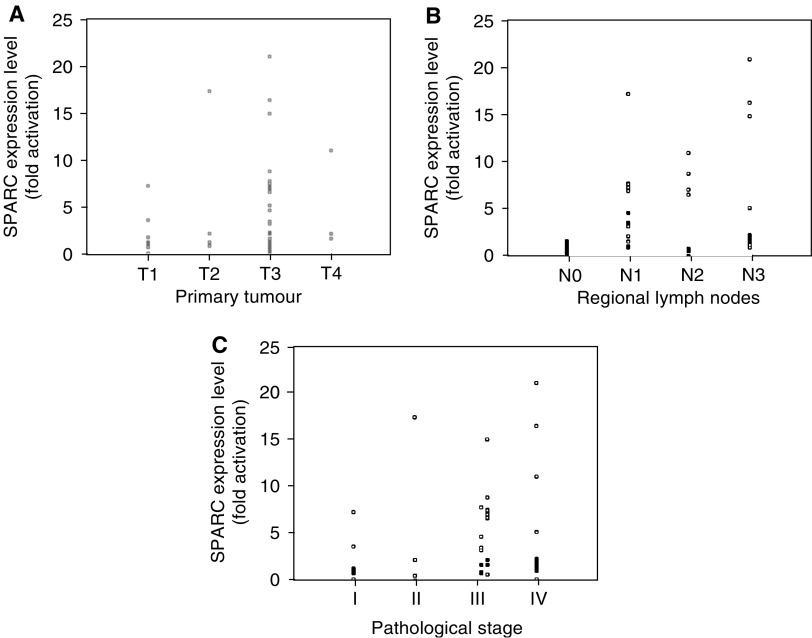
Scatterplots of comparison between the activation of SPARC and various clinicopathological features according to: (**A**) depth of wall invasion (*P*=0.048, T1 *vs* T2–4); (**B**) status of lymph node metastasis (*P*<0.001, N0 *vs* N1–3); (**C**) pathologic stage (*P*=0.017, stage I *vs* stage II–IV).

SPARC expression in tumour tissue was not significantly associated with age, gender, tumour location, tumour size, gross type or histological type (Table 1). Higher levels of SPARC were noted in advanced (T2, T3 and T4) cancer compared to the early (T1) cancer (*P*=0.048) with regard to depth of wall invasion (Figure 3A

). Higher expression of SPARC was significantly associated with lymph node metastasis (*P*<0.001) (Figure 3B), lymphatic invasion (*P*=0.004) and perineural invasion (*P*=0.047). It was not associated with vascular invasion or peritoneal seeding. Expression of SPARC in patients with pathologic stage⩾II was significantly higher than those with stage I (*P*=0.017) (Figure 3C).

### Survival outcome

**Figure 4:**
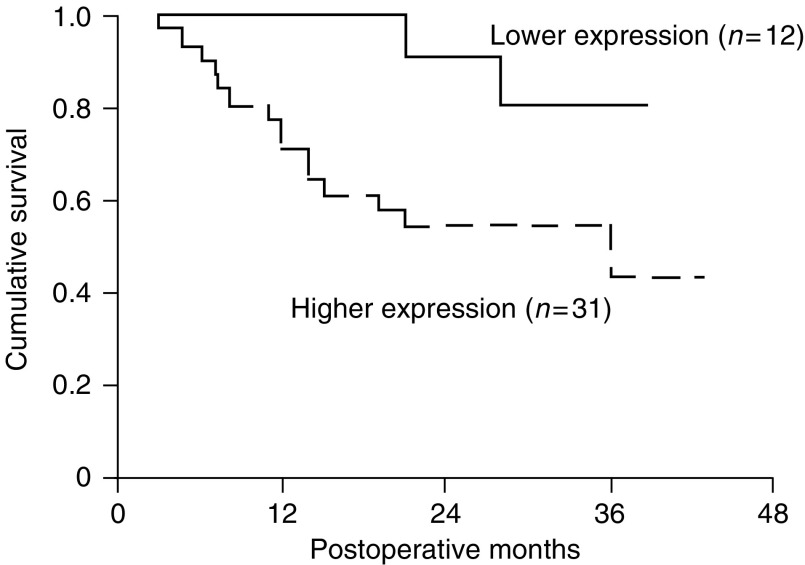
The Kaplan–Meirs survival curves of two groups of gastric cancer patients defined by a SPARC expression level cutoff value of 1.0, as determined by real-time Q-RT–PCR. The 3-year survival rate of the low expression group (⩽1.0-fold) in our patients was significantly better than that of the higher expression groups (>1.0-fold, 80.8 *vs* 43.7%; log rank *P*=0.047).

The mean duration of the follow-up period for survivors (*n*=26) was 30.5. months (range 20–43 months). In all, 17 patients died as a result of the progression of gastric cancer, and another patient died due to surgical complications. The overall cumulative 3-year survival rate of the 43 patients with gastric resection was 53.9%. Figure 4

illustrates the cumulative survival curves of patients divided into the lower expression (*n*=12) and higher expression (*n*=31) groups of SPARC. The lower expression group was defined as those with equal or lower (⩽1.0-fold) SPARC expression in the tumour, whereas the higher expression group consisted of patients expressing higher levels (>1.0-fold) of SPARC in the tumour than in the adjacent normal tissue, as determined by real-time Q-RT–PCR. The 3-year survival rate of the low expression group in our patients was significantly better than that of the higher expression groups (80.8 *vs* 43.7%; log rank *P*=0.047) (Figure 4).

Univariate analysis showed that other significant prognostic factors included the status of lymph node metastasis (N0/N1, N2, N3; log rank *P*<0.0001), serosal invasion (positive/negative; log rank *P*=0.0145), lymphatic invasion (positive/negative; log rank *P*=0.0007), peritoneal seeding (positive/negative; log rank *P*=0.0003), perineural invasion (positive/negative; log rank *P*=0.0014) and tumour size (⩽4 cm/> 4 cm; log rank *P*=0.0006). Multivariate analysis revealed that the status of lymph node metastasis was the only significant prognostic factor for the survival of the patients (Figure 4).

## DISCUSSION

In this study, SPARC was identified, by cDNA microarray, as one of the most overexpressed genes in more than 60% of the gastric cancer patients included in this study. The overexpression of SPARC was verified by Northern blot analysis, real-time Q-RT–PCR and immunohistochemistry. Therefore, cDNA microarrays are a useful way to perform a large-scale analysis of the expression level of thousands of genes simultaneously (Schena *et al*, 1995). When used for studies on clinical samples, of normal *vs* diseased, it may lead to the identification of novel biomarkers for these diseases (Zweiger, 1999). The biomarkers may be candidates for establishing early diagnosis and designing therapeutic targets for specific diseases or cancers.

In the patients examined here, higher SPARC expression was significantly associated with tumour progression (lymph node metastasis, lymphatic and perineural invasion) and the advanced stages of gastric cancer. In addition, patients with lower SPARC expression had an improved prognosis. A similar clinic–pathologic relationship between SPARC expression and tumour progression has been reported in other malignancies (Porte *et al*, 1995; Massi *et al*, 1999; Thomas *et al*, 2000; Sakai *et al*, 2001; Yamanaka *et al*, 2001; Yamashita *et al*, 2003). The expression of SPARC has been positively correlated with the histological grade of tumour cells in bladder cancer, thyroid cancer, glioma and HCC (Le Bail *et al*, 1999; Menon *et al*, 2000; Takano *et al*, 2000; Yamanaka *et al*, 2001). Higher SPARC expression was also associated with local tumour invasion in colorectal, breast, bladder and renal cell carcinoma (Porte *et al*, 1995; Sakai *et al*, 2001; Yamanaka *et al*, 2001; Iacobuzio-Donahue *et al*, 2002). Increased levels of SPARC were associated with lymph node metastasis in colorectal and oesophageal cancers, liver metastasis in colorectal cancer and bone metastasis in prostate cancers (Porte *et al*, 1995; Thomas *et al*, 2000; Yamashita *et al*, 2003). In addition, high SPARC expression was associated with poor prognosis and survival in colorectal cancer, melanoma and oesophageal cancer (Porte *et al*, 1995; Massi *et al*, 1999; Thomas *et al*, 2000). However, the expression is inversely correlated with the degree of ovarian malignancy, where the downregulation of SPARC is essential for carcinogenesis (Paley *et al*, 2000).

The cellular origin of SPARC is variable among malignant tumours. SPARC may be expressed predominantly either in the tumour cells or in the stromal cells depending on the types of malignancies. The protein immunoreactivity of SPARC was detected mainly in the stroma cells, but rarely in the tumour cells in malignant ovaries, colorectal cancer, breast cancer and hepatocellular carcinoma (Porte *et al*, 1995; Le Bail *et al*, 1999; Paley *et al*, 2000; Iacobuzio-Donahue *et al*, 2002). The signals originating from tumour cells regulate SPARC expression in neighbouring fibroblasts. When SPARC is secreted from stroma cells, it exerts its role on neoplastic progression in a paracrine fashion (Porte *et al*, 1995; Le Bail *et al*, 1999). In contrast, immunostaining for SPARC was located mainly in the tumour cells in melanoma cells and oesophageal cancer (Massi *et al*, 1999; Yamashita *et al*, 2003). In the gastric cancer patients included in this study, the SPARC expression was similar to that of oesophageal cancer, detected mainly in the gastric cancer cells, and less intensively in the stroma cells. Our finding was similar to that in one report on gastric cancer study (Wewer *et al*, 1988), but different from another report, where SPARC stained only the stroma cells in gastric cancer tissues (Maeng *et al*, 2002b).

Invasion and metastasis are the inherent characteristics of malignant diseases and involve both intercellular and cell–matrix interactions. The molecular mechanisms include the transcriptional modulation of adhesive and antiadhesive molecules, proteases and angiogenic factors. It has been suggested that SPARC may play a key role during the initial steps in the process of tumour invasion and metastasis (Porte *et al*, 1995). One of SPARC's biological functions is the modulation of angiogenesis (Lane *et al*, 1994). It may promote neo-vascularisation, invasion and metastasis of human malignancies (Motamed, 1999; Paley *et al*, 2000; Thomas *et al*, 2000; Sakai *et al*, 2001). Furthermore, SPARC has demonstrated antiadhesive properties and displayed diminished adhesive interactions between tumour cells and the extracellular matrix via the reduction of cell–substrate contacts and the promotion of cytoskeletal rearrangement (Ledda *et al*, 1997). The antiadhesive properties of SPARC facilitate the invasion and metastasis of tumour cells (Thomas *et al*, 2000). In addition, SPARC can induce the expression of metalloproteinases or enzymes that subsequently play an important role in the degradation of basal membranes and extracellular matrix components (Tremble *et al*, 1993). A significant correlation with MMP-2 gene expression implies that the regulation of MMP-2 may be a possible mechanism underlying the effect of SPARC on the progression of oesophageal and bladder cancer (Ledda *et al*, 1997; Thomas *et al*, 2000; Yamanaka *et al*, 2001; Yamashita *et al*, 2003).

Our work defines the mis-regulation of SPARC in gastric carcinoma and its strong association with the more advanced stages of this disease. The overexpression of SPARC in a variety of malignancies excludes it as a suitable diagnostic biomarker for any specific tumour, although it displays promise as a prognostic marker of tumour progression and advanced cancer stage. We had analysed the serum SPARC expression in 20 gastric cancer patients by the Western blot. However, we did not detect the expression of SPARC under our experimental condition (data not shown). However, the serum concentration of SPARC had been detected by a sandwich ELISA method in the type II diabetes mellitus patients (Kanauchi *et al*, 2000). Whether determination of the serum SPARC level can be used in clinical diagnosis awaits further study.

The identification of the molecular determinants of invasion and metastasis will guide the development of new therapies. Two recent *in vitro* studies delineate the potential of SPARC as a suppressor of tumorigenic potential in human melanoma and breast cancer cells via the addition of either antisense RNA or the transfection of SPARC (Ledda *et al*, 1997; Dhanesuan *et al*, 2002). Finally, our study demonstrates that the further investigation of SPARC is warranted due to its status as a potential prognostic and therapeutic agent.
